# Learning of efficient behaviour in spatial exploration through observation of behaviour of conspecific in laboratory rats

**DOI:** 10.1098/rsos.170121

**Published:** 2017-09-20

**Authors:** Yuji Takano, Masatoshi Ukezono, Satoshi F. Nakashima, Nobuaki Takahashi, Naoyuki Hironaka

**Affiliations:** 1 Center for Baby Science, Organization for Research Initiatives and Development, Doshisha University, 4-1-1, Kizugawadai, Kizugawa, Kyoto 619-0225, Japan; 2 CREST, JST, Atsugi, Japan; 3 Department of Neuropsychopharmacology, National Institute of Mental Health, National Center of Neurology and Psychiatry, Tokyo, Japan; 4 NTT Communication Science Laboratories, Nippon Telegraph and Telephone Corporation, Atsugi, Japan; 5 Department of Clinical Psychology, Bukkyo University, Kyoto, Japan; 6 Department of Pharmacology, LSI Medience Corp., Tokyo, Japan

**Keywords:** observational learning, social facilitation, spatial memory, rat

## Abstract

Recent studies have suggested that rodent behaviour is influenced by the behaviour of surrounding conspecifics (e.g. emotional contagion and prosocial behaviour). However, little is known about deferred imitation and complex observational learning in rats. The purpose of this study was to reveal whether rats can learn from another rat's experiences. In a maze, observer rats watched the foraging behaviour of other rats (demonstrators) and then foraged in turn. The results showed that demonstrators explored inefficiently, but observers explored more efficiently after observing inefficient exploration by the demonstrators. This observational learning probably involved the acquisition of an efficient strategy through spatial exploration.

## 1. Introduction

Learning from others is an important cognitive function for survival in social animals. If one cannot learn that inefficient strategies bring no reward after observing them, then energy will be wasted. As a result, no reward will be received by the non-learner, whereas those with the capacity to learn from observing others will receive more than their fair share of reward.

Many primate species, such as humans, chimpanzees and monkeys, can learn efficient strategies from others [1–5]. Investigating social learning in primates involves examining skill acquisition by an observer to acquire novel skills and understand causal relationships based on observation of the demonstrator's behaviour. Most studies have focused on whether the observer imitates the demonstrator's successful behaviour [6,7].

Little is known, however, about complex social learning in mammals other than primates, such as rodents. Some studies have shown that rodents use information obtained from other conspecifics. For example, food preferences are socially transmitted in rats [8–10]. After they interact with a demonstrator rat, observer rats prefer unfamiliar foods that the demonstrator has eaten [8]. Relationships between observers and demonstrators, such as kinship, familiarity and dominance, modulate the social transmission of food preference [11,12].

In addition, some behaviours in rats are socially facilitated by the presence of other conspecifics [13]. Harlow showed that rats eat more food when they eat with other conspecifics than when they eat alone [14]. This implies that rat behaviour is affected by the presence of co-acting conspecifics. Moreover, we have previously shown that the presence of other conspecifics is sufficient to influence rat behaviour. We found that spin behaviour was faster before reaching food in the presence of other conspecifics [15]. These findings suggest that rats change their behaviour based on their social context.

Prosocial behaviour in rats may influence their behaviour based on the situation of other conspecifics. Viana *et al*. showed that rats work cooperatively in the human prisoner dilemma model [16]. Ben-Ami Bartel *et al*. showed that rats help other rats restrained in a tube [17]. In addition, Sato *et al*. reported that rats help other rats who are soaked [18]. These studies suggest that rodents behave prosocially (e.g. they cooperate and help) towards other conspecifics.

Rodents may adjust their own behaviour according to the situation of other conspecifics, particularly using visual information. Langford *et al*. showed that mice display greater pain behaviour after suffering painful stimuli together with other conspecifics than they do alone [19]. This indicates emotional contagion among rodents. Importantly, socially mediated hyperalgesia was attributed to visual information from other conspecifics. In this context, we examined whether rats can receive emotional signals visually from other conspecifics [20]. We measured whether rats prefer images of conspecifics with a neutral expression or an expression of pain. Rats were able to discriminate expressions of pain from neutral expressions. In addition, they actively avoided pictures showing an expression of pain. This indicates that rats can judge the situation of other conspecifics and adjust their own behaviour to avoid negative outcomes.

**Figure 1.**
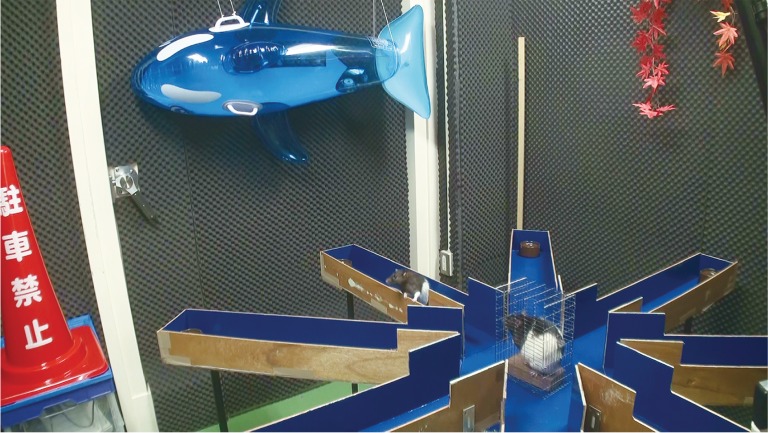
Experimental set-up: Normal eight-arm radial maze with an observation room in the centre arena. In this scene, a demonstrator rat is exploring the maze in front of an observer rat. After the demonstrator rat has explored, the observer rat starts to explore.

Although rodents may be able to assess the situation of others, whether they can learn efficient strategies based on the behaviour of others has not been elucidated. This study investigated whether rats can learn efficient strategies from the experiences of conspecifics. We developed a new task to measure observational learning in rats. An eight-arm radial maze was used. Such radial mazes are based on natural foraging activities of rats and used to measure their spatial memory [21]. We prepared a closed observation room in the centre of the maze (figure 1). Four of the eight arms were baited. After one rat (observer) was put in the centre room, another rat (demonstrator) was trained to find food pellets in the maze. After the demonstrator found all the food pellets, the apparatus was cleaned and food pellets were placed in the same bait trays as before. Then, the observer was allowed to explore. This task allowed us to examine whether the presence of the observer affected the behaviour of the demonstrator and whether the behaviour of the demonstrator affected the exploration behaviour of the observer. We predicted that observer rats would find the food rewards faster than the demonstrator rats or control rats that did not observe the behaviour of other conspecifics. This demonstrates that rats can learn efficient strategy from other rats.

## 2. Methods

### 2.1. Animals

In all, 56 male Long–Evans rats (S.L.C. Japan Inc.) were used and were 5 weeks old at the time of purchase. Rats were randomly allocated into pairs and housed in a temperature-controlled (22–23°C) room under a 12 h light–dark cycle (lights on from 08.00 to 20.00). The rats had free access to laboratory chow (CE-2, CLEA Japan Inc.) and tap water in their home cages during the first 2 weeks. Each cage was provided with 33 g laboratory chow per day. The day before the experiment, approximately 6 g laboratory chow and 14 g sucrose pellets were given to each cage. Tap water was freely available in the home cages at all times. We divided the rats randomly into the following four groups: demonstrator, observer, waiting and individual. The demonstrators and observers were cage mates. In the waiting and individual groups, we allocated pairs of rats from the same cages to the same groups. We started to train the animals 5 weeks after purchase, so that the animals were 10 weeks old at the beginning of the experiment. There were 14 rats each in the demonstrator, observer, individual and waiting groups.

### 2.2. Apparatus

The eight-armed radial maze was made of wood and consisted of two parts. One was the centre arena, which was an equilateral octagon-shaped space with a diameter of 36 cm. A wire-mesh cage (15 × 11 × 23 cm) was placed in the centre (the observation room) (figure 1). The entrance to the wire-mesh cage was directed toward the entrance of the experimentation room, and the orientation of the cage was constant across trials. The other part was eight 80 cm arms, each with a guardrail. The height of the guardrails in the binding sites between each arm and the centre arena was 14 cm and that of the guardrails in the remaining sites of arms was 9.5 cm. The apparatus was high-floored, and the floor-to-apparatus height was 72 cm. There were bait trays at the end of the arms. We put four sucrose pellets each in four of the eight bait trays in the maze (60 mg per tray). These arms were called correct arms. Some objects were arranged around the apparatus with north, south, east and west orientations. The animals' behaviours were recorded with a video camera.

### 2.3. Training

We put sucrose pellets in the correct arms. The correct arms were always the same four arms for each rat during the 30 trials.

Then we placed a rat on the side of the observation room in the centre of the arena and removed it after it had visited the four correct arms and returned to the centre arena. For all groups, each rat was put in the centre of the arena with the direction of the entrance of the observation room. There was no time restriction. After each rat was removed, we cleaned the floor and guardrails in the maze with a wet paper towel. This constituted a trial, and we conducted one trial per day and six trials per week, for a total of 30 trials over 5 weeks. The above schedule was followed in training individual groups.

In the demonstrator and observer trials, we put an observer rat into the observation room from the entrance after putting pellets into the four bait trays. The observer rats could change direction at any time in the observation room. Then we placed a rat from the demonstrator group in the centre arena. The demonstrator rat was removed after it returned to the centre arena after visiting the correct arms. After sweeping the maze and restocking the pellets in the same trays, we brought the observer out from the observation room and placed it in the centre of the arena, as was done with the demonstrator. The demonstrator trial preceded the observer trial for each pair, on the same day. The observation room was kept at the centre of the arena during the observers' trials, as was the case with the demonstrators' trials, though the inside of the cage was empty.

There were two control groups: individual and waiting. Both control groups explored the maze without any observation beforehand. The waiting group explored after spending an equal amount of time in the observation room in common as the observer rats did, although without a demonstrator. The waiting group was used to rule out the possibility that the observer's performance was dependent on habituation to the maze. Rats in the individual group were allowed to explore without waiting. The observation room was kept at the centre of the arena, as was done with other groups, and the inside of the cage was empty during trials.

### 2.4. Data analysis

We recorded the time taken to complete one trial and the number of arm choices before the four correct arms were visited. An arm choice was defined as the rat reaching the bait tray at the end of the arm. We considered transit time to be the duration required by the animals to visit the four correct arms, starting from the start of the trial; this was an index of performance assessment. We divided the transit time by the total number of arm choices. The resulting value was the searching inefficiency.

**Figure 2.**
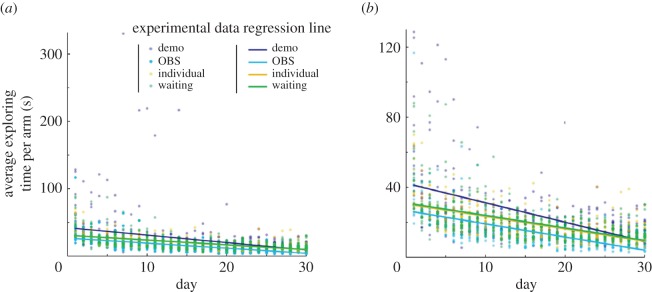
Learning curves of the average time taken to explore each arm by trial and regression lines for each group. We show an extended figure (*b*) that gives the main parts of the data from the entire dataset (*a*) to clearly show the trends of each condition. We used this daily trial data to conduct multiple regression analysis. Demo: demonstrator group; OBS: observer group; individual: individual group; waiting: waiting group.

To examine differences in searching inefficiency among the conditions throughout the course of the trials (figure 2; electronic supplementary material, table S1), we conducted a hierarchical regression analysis on the index of searching inefficiency. We regressed searching inefficiency according to trial number, dummy variables for each condition and the interaction term of trial number and dummy variables for each condition. For the analysis, we created three dummy variables corresponding to each condition (for each variable, the demonstrator, observer or waiting group was assigned ‘1’, and the remaining group was assigned ‘0’: the individual group, as the control condition, was constantly assigned ‘0’). The interaction term between the trial number (1–30) and the dummy variables of each condition was computed by multiplying the trial number by each condition [22]. By adding the interaction terms using the regression formula, we could examine whether the effect of the time course of the trials on searching inefficiency (the time course of the variation of inefficiency through 30 days of training) was different for different values of each condition. This analysis was useful for comparing overall trends of change in each condition in searching inefficiency.

**Figure 3.**
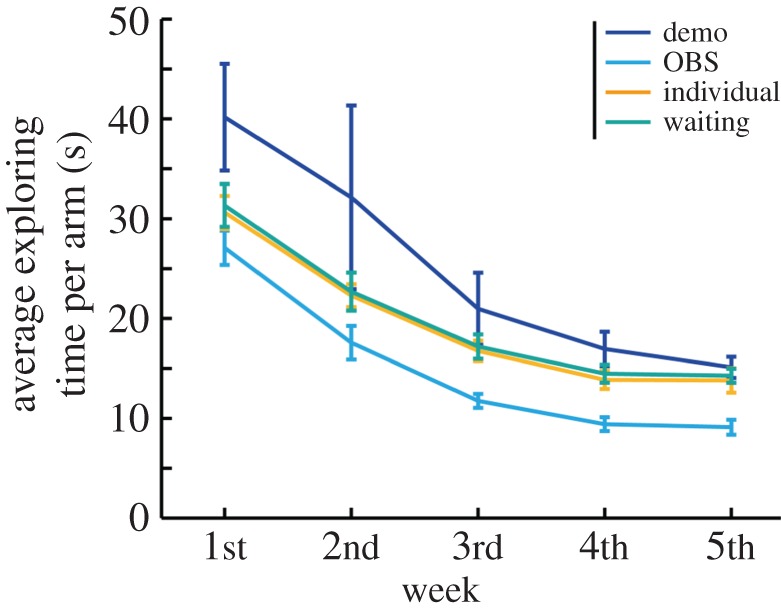
Learning curves of average time taken to explore per arm by week. We used this weekly averaged data to perform ANOVA.

**Figure 4.**
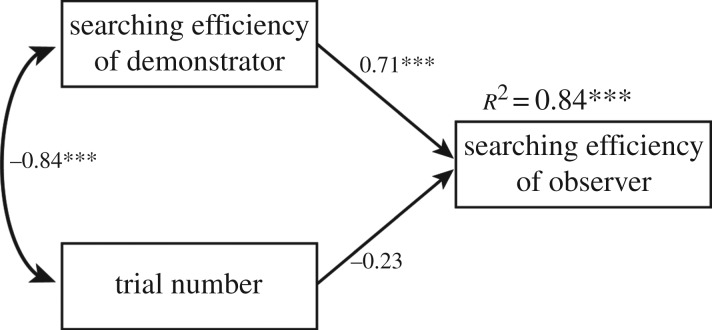
Path diagram representing the effect of observing the demonstrator's behaviour on the searching inefficiency of observers. The numerical values represent standardized partial regression coefficients. The term ‘searching inefficiency’ means the average time to explore one arm. The greater this value is, the less efficiently the rat collected the pellets. ***p < 0.001.

To determine when differences in searching inefficiency occurred among the conditions, we averaged inefficiency over 6 days, plotted learning curves and analysed the data using one-way ANOVA within the four groups (figure 3). We used Holm's correction as a *post hoc* analysis. The results for other indices, transit time and the number of errors (entries of a non-baited arm and re-entries of an arm) are shown in electronic supplementary material, figure S1. To test the relationship between the searching inefficiency of the demonstrator and observer, we performed additional regression analyses of searching inefficiency. We regressed observer searching inefficiency by trial number and demonstrator searching inefficiency (figure 4). In addition, we tested the model using transit time or the total number of errors to compare the prediction power (*R*^2^) of each model (electronic supplementary material, figure S2).

Although the results in searching inefficiency might imply that rats in some conditions show efficient behaviour, it does not actually suggest which behaviour makes the difference for each condition. We therefore additionally assessed whether the rats in each condition showed different exploring behaviours. For this purpose, we focused on rearing (standing upright on the hind legs), which is related to vigilance, arousal and exploring, during searches in each arm. We considered that the number of rearings per arm could be an index of the inefficient strategic behaviour of rats. An index of inefficient strategic behaviour was calculated by dividing the total number of rearings during exploration in each arm by the total number of arm choices. We counted the number of rearings, defined as when the forepaw of the rat reached higher than the outside wall of each arm after the hind legs of rat moved. We did not count as rearings movements such as taking a look around without the movement of the hind legs. In calculating the index of inefficient strategic behaviour, we used the averaged data of the fifth week, when the rats were well trained. The higher index of inefficient strategic behaviour means that rats showed more wasteful behaviours inside of each arm.

## 3. Results

We recorded the number of errors (entries to the non-baited arm and re-entries to the arm) and the time to find all pellets and then calculated the average exploration time per arm (named inefficiency). The greater this value is, the more poorly the rat collected the pellets.

First, to examine whether the variability with the time course in searching inefficiency is different between each condition, we conducted hierarchical regression analyses on the inefficiency data. Regression lines of the time-series variation of searching inefficiency for each condition are depicted in figure 2. We regressed the inefficiency by trial number, dummy variables for each condition (demonstrator, observer and waiting) and interaction between trial number and each dummy variable (electronic supplementary material, table S1). In model 1, inefficiency was regressed by the trial number and dummy variables for each condition. Results showed that trial number correlated negatively with inefficiency (*b* = −0.82, s.e. = 0.04, *t*_1675_ = −18.57, *p* < 0.001), suggesting that searching inefficiency decreased as the trials progressed. The dummy variable for the demonstrator correlated positively with inefficiency (*b* = 5.59, s.e. = 1.08, *t*_1675_ = 5.15, *p* < 0.001), whereas the dummy variable for the observer correlated negatively with inefficiency (*b* = −4.49, s.e. = 1.08, *t*_1675_ = −4.14, *p* < 0.001). The dummy variable for the waiting group was not significant (*b* = 0.51, s.e. = 1.08, *t*_1675_ = 0.47, n.s*.*)*.*

These results indicated that the observer group learned more efficiently than the individual group, whereas the demonstrator group learned less efficiently than the individual group. This explained approximately 20.5% of the variance in inefficiency [*R*^2^ = 0.205, *F*_4, 1675_ = 107.84, *p* < 0.001]. In model 2, we tested the prediction that the increment of searching inefficiency throughout the course of the trials was different between conditions. There was a significant interaction between trial number and demonstrator (*b* = −0.40, s.e. = 0.13, *t*_1672_ = −3.21, *p* < 0.01). In contrast, no other interactions were significant. The results indicated that the slope of the regression line for the inefficiency throughout the course of the trials was different between individual and demonstrator rats. However, no difference was observed between the individual group and all other groups. This explained 0.7% of the incremental variance in searching inefficiency (Δ*R*^2^ = 0.007, *p* < 0.01) and yielded an *R*^2^ of 0.212 for the model overall (*R*^2^ = 0.212, *F*_7, 1672_ = 64.12, *p* < 0.001). Simple slope analysis [20] showed that the regression line of inefficiency throughout the course of the trials in the demonstrator group was sharper than that in the individual group (demonstrator: *b* = −1.11, s.e. = 0.09, *t*_1672_ = −12.59, *p* < 0.001; Individual: *b* = −0.71, s.e. = 0.09, *t*_1672_ = −8.05, *p* < 0.001). The results indicated that the learning progress in the demonstrator group was different from that in individual and other groups and that learning progress of demonstrators exhibited more drastic changes than those in other groups.

Second, we examined when differences in searching inefficiency between conditions occurred specifically. The weekly average inefficiency was analysed by ANOVA with Holm's multiple comparison (figure 3). In the first week, there was a significant difference between groups (*F*_3, 55_ = 2.95, *p* < 0.05, *η^2^* = 0.15). The difference between the inefficiency of the observer group and the demonstrator group (*p* < 0.05) was significant. Although the overall group difference was not significant in the second week (*F*_3, 55_ = 1.50, n.s*.*, *η^2^* = 0.08), significant group differences were observed again in the third week (*F*_3, 55_ = 3.30, *p* < 0.05, *η^2^* = 0.16), and the inefficiency was significantly different between the observer and demonstrator groups (*p* < 0.05). In the fourth and fifth weeks, there were significant differences between groups (*F*_3, 55_ = 7.24 and 7.35, both *p* < 0.001, *η^2^* = 0.29 and 0.30, respectively). Significant differences were observed between the observer and demonstrator groups (week 4: *p* < 0.01; week 5: *p* < 0.01), observer and individual groups (week 4: *p* < 0.05; week 5: *p* < 0.01) and observer and waiting groups (week 4: *p* < 0.05; week 5: *p* < 0.01). In summary, the efficiency of foraging was highest in the observer group throughout the course of learning and lowest in the demonstrator group, particularly in the early phase of training.

Third, to test the possibility that the searching inefficiency of observers was affected by observing the performance of demonstrators, we conducted an additional regression analysis on the searching inefficiency of observers (figure 4). We regressed the observer inefficiency by trial number and demonstrator inefficiency. The results showed that the demonstrator inefficiency was positively related to the observer inefficiency, (*b* = 0.49, *B* = 0.71, s.e. = 0.10, *t*_27_ = 5.04, *p* < 0.001). In contrast, the effect of trial number was not significant, (*b* = −0.21, *B* = −0.23, s.e. = 0.13, *t*_27_ = −1.66)*.* This model explains approximately 84% of the variance in observer searching inefficiency (*R*^2^ = 0.84, *F*_2, 27_ = 71.12, *p* < 0.001). This indicates that the searching inefficiency of demonstrators still affected the searching inefficiency of observers, even when the effect of the time-series variation of learning was statistically controlled. Therefore, we believe that the relationships between demonstrator and observer inefficiency were not invalid or caused by learning over time. In addition, we confirmed that the model based on inefficiency is better than the one based on total transit time or total errors, according to the *R*^2^ values of each model (electronic supplementary material, figure S2).

**Figure 5.**
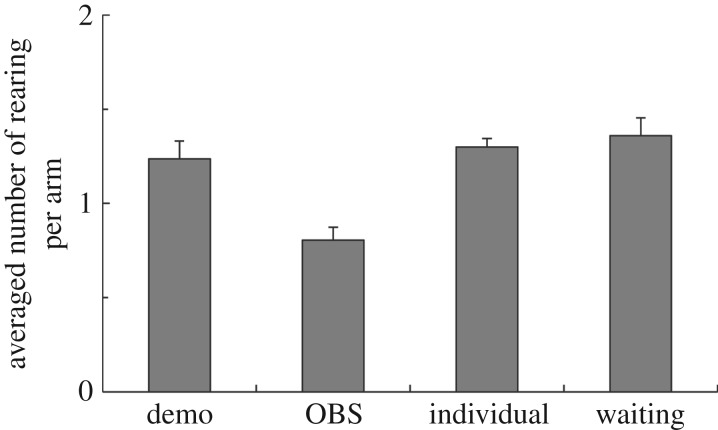
Averaged number of rearing per one arm at 5th week. The average number of rearing per one arm in observer was lower than all of other conditions (all *p*s < 0.01). In contrast, there were no significant differences between any other conditions (n.s.).

Finally, we examined whether inefficient strategic behaviour during exploring was different between each condition by comparing the number of rearings per arm. The average number of rearing per one arm was analysed by ANOVA with Holm's multiple comparison (figure 5). There was a significant difference between conditions (*F*_3, 55_ = 10.16, *p* < 0.001, *η*^2^ = 0.37). The average number of rearing per one arm in observer was lower than all of other conditions (all *p*s < 0.01). In contrast, there were no significant differences between any other conditions (n.s*.*).

## 4. Discussion

This study determined whether rats can learn efficient strategies from the experience of other rats. For this purpose, we developed a new task to measure observational learning. A demonstrator rat explored a maze to find food rewards in the presence of an observer rat. Then the observer rat performed the task. We hypothesized that the observer rats would find the rewards faster than the demonstrator rats and the other control rats, demonstrating that rats can learn efficient strategies from the behaviour of others.

Our hypothesis was correct. Throughout the experimental period, observer rats learned more efficiently than the rats in other conditions. Searching inefficiency was not significantly different between individual and waiting rats, indicating that behavioural differences were not attributable to habituation to the maze. Therefore, we consider that the efficiency of observer behaviour was at least partly derived from the presence of the demonstrator. The searching inefficiency of the observer correlated positively with that of the demonstrator, even after excluding the effects of variation during learning. This indicates that observer rats improve searching efficiency by observing demonstrators. The presence of other conspecifics has been shown to stimulate many behaviours in rats, including social facilitation [13] and the social transmission of food preference [8]. However, a relationship between demonstrator behaviour and observer behaviour has not been previously shown. In this study, we presented new evidence that the behaviour of demonstrators affects that of observers.

For the searching efficiency in the behaviour of an observer, we conducted additional analysis on the average number of rearings per arm as an index of inefficient strategic behaviour in the period after being well-trained. In the results, we found significant differences between the observer condition and all others but did not find any differences between the demonstrator and control conditions. This result means that the observer behaved efficiently by quickly receiving the necessary environmental information to obtain rewards without wasteful behaviour. Importantly, although rats in all other conditions were already well-trained, there were still significant differences in efficient behaviour (see electronic supplementary material, film S1).

In addition, regression analysis showed that demonstrators learned less efficiently than rats in the individual and waiting groups. The learning curve was steeper in demonstrator rats than in control rats, and the greatest difference was observed during the initial stages of learning. These findings indicate that the demonstrators were distracted from learning by the presence of the observer during the initial stages of learning. Their searching efficiency reached the levels of the searching efficiency of rats in the individual condition in the later stages of learning.

Interestingly, our findings show that observational learning may not have been due to simple copying actions. Demonstrator rats were inefficient at finding food rewards during the early phases of learning. Therefore, if the observer rats had simply copied the actions of the demonstrator, they would have displayed a low searching efficiency during the early stages of learning. However, observer rats behaved more efficiently than other rats during all stages of learning. Additionally, the analysis of inefficient strategic behaviour implied that observer rats acquired spatial information more quickly than rats in all other conditions, though demonstrator rats did not. This suggests that rats learn efficient strategies by observing inefficient behaviour. This implication is in line with previous findings that correct responses are promoted by observing incorrect answers given by others [23].

With regard to this point, it might be possible that the observer adopted efficient strategic behaviour as a consequence of learning about the safety of the experimental environment by observing the behaviour, rather than learning the precise behaviour of the demonstrator. A radial maze is an inherently unfavourable environment for rats, because the inside of the maze has good sight visibility and there is no place to hide. It is, therefore, undeniable that the rats may have learned information on safety by observing the behaviour of the demonstrator, although even in this case, the observer may have learned some sort of efficient strategic behaviour by observing the behaviour of the demonstrator. On the other hand, learning the safety of the environment is difficult for rats. In the study, the observer showed a greater searching efficiency than the other conditions, although the searching efficiency of the waiting condition, which was habituated to the environment, was not different from that of the individual condition. From this point of view, another intriguing possibility is that learning of the safety of the unfavourable environment, a difficult task for rats, was facilitated by observing other conspecifics. Further studies should examine this possibility.

This study may provide a new perspective on the evolution of observational learning. Traditionally, complex social learning such as observational learning has been thought to have evolved only in primate species (e.g. [1–5]). Thus, limited attention has been paid to observational learning mechanisms in rodents. Our findings indicate that observational learning mechanisms are also present in rodents, although the specific mechanisms remain unclear.

There is another possible explanation for the increased search efficiency of observer rats. Observer rats may simply have used the positions of demonstrator rats as spatial cues to find food pellets [24]. However, this is unlikely, because the average rates of correction were the same in observers and demonstrators; therefore, the observer did not memorize a simple association between the position of a demonstrator and the presence of reward at the position. In other words, rats may not learn from other rats' experience. Observational learning may also have been influenced by social facilitation mechanisms. Zajonc showed that performance in familiar tasks, but not novel tasks, was facilitated by the company of others [25]. The social facilitation effect is thought to increase arousal in front of others, promoting searching efficiency. The poor searching efficiency of the demonstrators in the early phase may be explained by social inhibition effects; the demonstrator rats were inhibited by the presence of the observer before they were accustomed enough to the maze to forage in it. However, it is difficult to explain the later differences in foraging behaviours using social facilitation, because both the demonstrators and observers were accustomed to foraging in the maze.

We will also mention another possible reason why the demonstrator showed poorer searching efficiency than other conditions: the social buffering effect. Social buffering occurs when the presence of and the interaction with other conspecifics diminish the stress level in mammals, such humans, monkeys, rats and pigs (e.g. [26–29]). Several studies showed that stress levels in rats that were highly stressed, as measured by corticosterone and prolactin, decreased after interaction with other conspecifics (e.g. [30,31]). In our study, observer rats were in a cage and might have been stressed, though there was no stress promoter as in previous studies (e.g. foot shock), and they could minimally interact with the demonstrator with their nose through the small holes of the central cage (figure 1). In fact, especially in the early phase of learning, the demonstrator rats actively interacted with the observer rats in the centre arena. The demonstrators were possibly, therefore, distracted from their searching behaviour through allocating their attention to the observer rats, especially during the early phase of learning. Examining the influence of social buffering on the behaviour of the demonstrator is important for revealing the mechanism of the behaviour of the demonstrator when the observer is present. Future studies should examine whether social buffering affects the behaviour of the demonstrator in an organized manner.

On the other hand, it is also considered that the behaviour of the observers might be affected somewhat by the social buffering effect. From the social buffering effect, observer's stress was diminished by the interaction with demonstrator. The stress level in the observers, therefore, might decrease and their searching behaviour might be affected by the stress level. In this regard, however, only the social buffering effect is difficult to explain the efficient searching behaviour of observer, because the results revealed that the observer showed the highest searching efficiency even in the later stage of learning. In the later stage of learning, observer rats were already highly habituated to their situation and did not feel much stress, so that it is highly unlikely that social buffering affected the behaviour of the observer. As noted earlier, crucially, the searching efficiency in the observer was superior to that of rats of any other control conditions that had no stress. In addition, the results of regression analysis showed that the inefficiency of demonstrator significantly predicted the inefficiency of observer, even after controlling time-series variation. This indicates at least that the observer learned efficient behaviour from the behaviour of the demonstrator. Taken together, it appears that the efficient behaviour of the observer derived from acquiring efficient strategic behaviour through observational learning, though the possibility is undeniable that the behaviour of the observer is somewhat affected by social facilitation and/or social buffering.

The observer condition in our study differed from that of previous studies in the time point at which conspecifics were present. In our study, the searching efficiency of the observer was facilitated after demonstrator rats finished the task and were taken away. In previous studies, task performance was facilitated concurrently with the presence of other conspecifics. Therefore, it may be difficult to directly compare the results of our observer condition with those of previous studies.

In addition, there is an important point to be emphasized in this context. In many previous studies, the demonstrator and observer rats conducted tasks concurrently, so the observer was not required to memorize the demonstrator's behavioural tendency (e.g. [24,32], but [33]). However, in our study, as noted above, there was a time delay between the demonstrator's performance of the task and that of the observer. The mechanism of the improvement of searching efficiency in observer, therefore, may be based on retaining the long-term memory of the behavioural tendencies of the demonstrator. Future studies should examine the facilitation phenomenon in our observer condition and those of previous studies.

We should also mention that there remains the question of the mechanisms of the findings of the study. Although we showed that observers improve their search efficiency by observing the behaviour of demonstrators, the kinds of demonstrator behaviours that improve searching efficiency in observer rats remain unclear. For instance, it is possible that the observers acted on the basis of several kinds of searching-relevant behaviours of a demonstrator, such as running, rearing and eating. Further studies are needed to explore the actual mechanisms of observational learning in rats.

In conclusion, this study demonstrated that rats learn efficient strategies by observing the behaviour of other rats, even when the behaviour is inefficient. This means that rats may use social information to extract efficient behaviour from other conspecifics, much more than previously thought. Additionally, our procedure for assessing observational learning in rats will be useful for neuroscientific and/or pharmacological research to explore the neural mechanisms of social information processing in rodents.

## Supplementary Material

Fig. S1

## Supplementary Material

Fig. S2

## Supplementary Material

Table S1

## Data accessibility

All data are available at Dryad (http://dx.doi.org/10.5061/dryad.3kg03) [34].
