# Supplementary figures and images for: Learning of efficient behaviour in spatial exploration through observation of behaviour of conspecific in laboratory rats
Source: R Soc Open Sci. 2017 Sep 20;4(9):170121. doi: 10.1098/rsos.170121 (PMC5627071; doi:10.1098/rsos.170121)

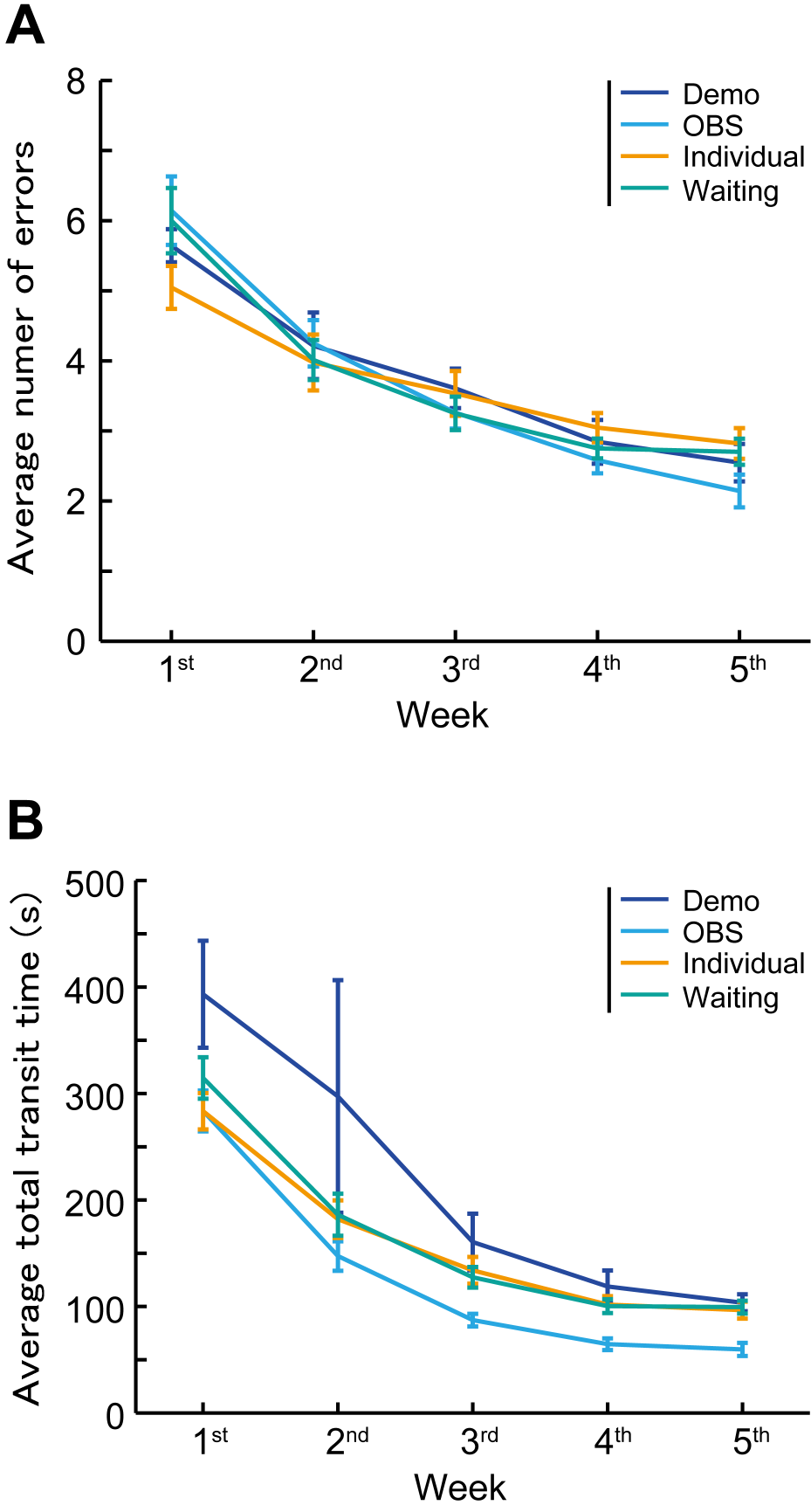

Supplement: Fig. S1 [file rsos170121supp1.tif]

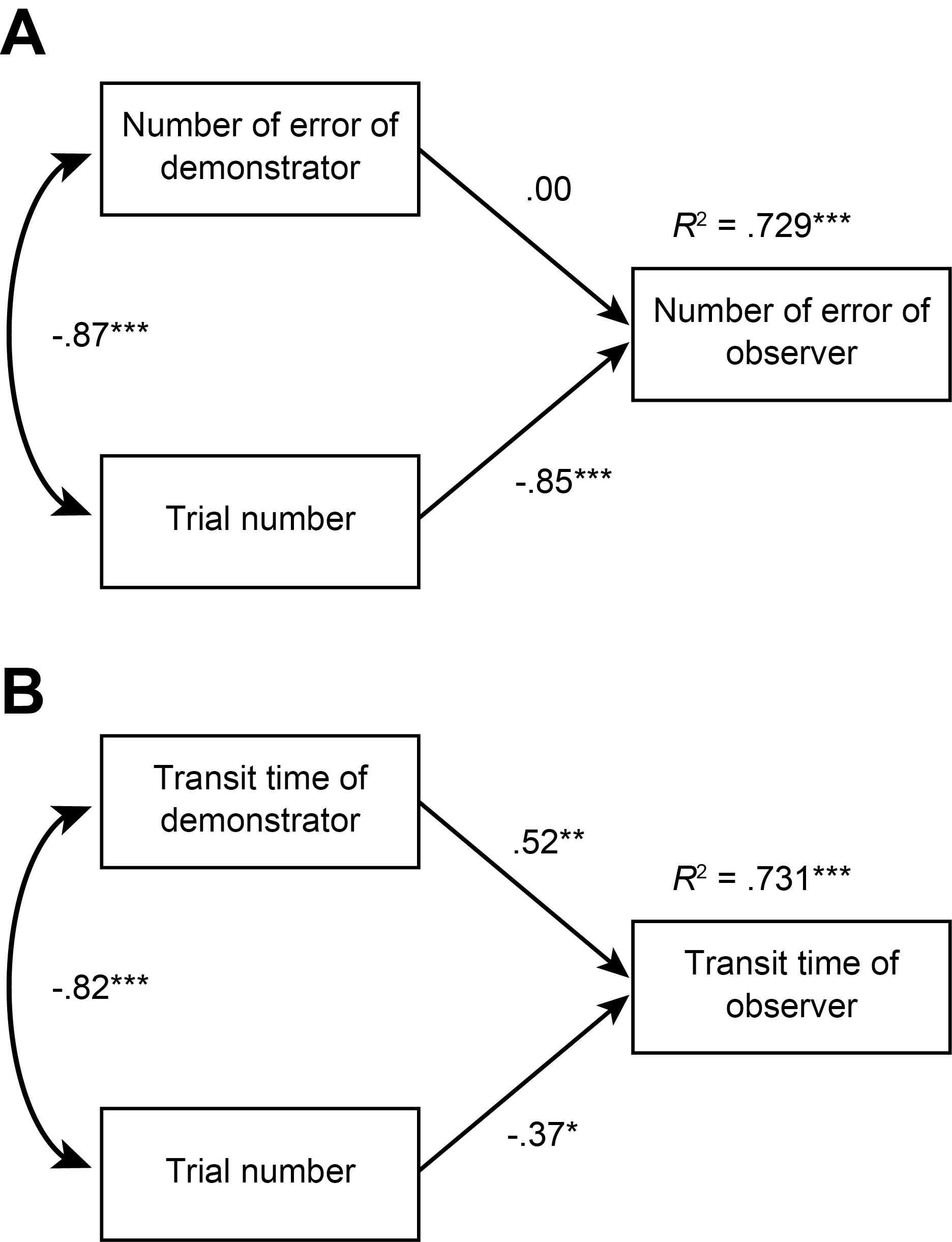

Supplement: Fig. S2 [file rsos170121supp2.tif]

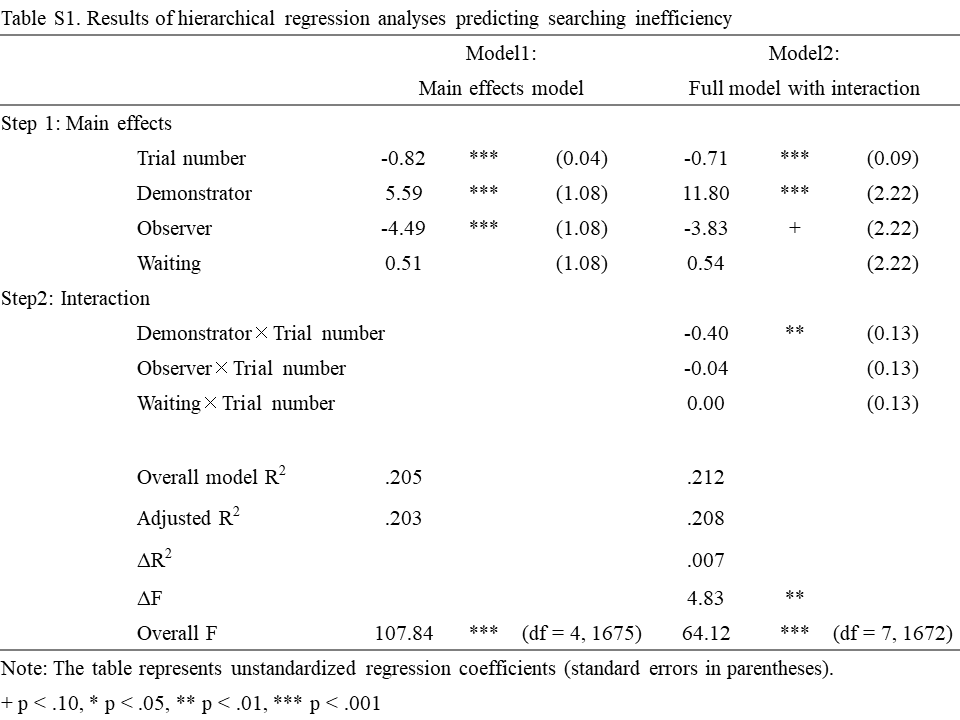

Supplement: Table S1 [file rsos170121supp3.tif]
